# Blood lactate as a marker of exercise adaptation and fatigue in male mice with chronic heart failure

**DOI:** 10.14814/phy2.70485

**Published:** 2025-07-30

**Authors:** Ahmed M. Wafi, Lie Gao, Irving H. Zucker

**Affiliations:** ^1^ Basic Medical Science Department, Faculty of Medicine Jazan University Jazan Saudi Arabia; ^2^ Department of Anesthesiology University of Nebraska Medical Center Omaha Nebraska USA; ^3^ Department of Cellular and Integrative Physiology University of Nebraska Medical Center Omaha Nebraska USA

**Keywords:** blood lactate, chronic heart failure, exercise training, fatigue, incremental exercise test

## Abstract

Treadmill exercise tests assess aerobic capacity in chronic heart failure (CHF) mice, but subjective exhaustion criteria limit reproducibility. Elevated blood lactate (La) at exhaustion may provide an objective fatigue marker. This study evaluated La at exhaustion and the effects of exercise training (ExT) on resting La in CHF mice. Sham and coronary artery ligation‐induced CHF mice were assigned to sedentary (Sed) or ExT groups. Blood La was measured from 0.7 μL tail vein samples at rest and at exhaustion during exercise tests after 8 weeks of ExT. Blood La at rest was slightly higher in CHF‐Sed mice (3.33 ± 1.23 mmol/L) than in Sham‐Sed mice (2.96 ± 0.42 mmol/L; *p* = 0.86). ExT slightly reduced La in CHF mice (2.55 ± 0.63 mmol/L; *p* = 0.34 vs. CHF‐Sed), but differences were not significant. At exhaustion, blood La increased significantly across groups. Baseline La in CHF‐Sed mice negatively correlated with running distance (*r* = −0.84, *p* = 0.03). Blood La levels at exhaustion offer a cost‐effective method to confirm exhaustion criteria and enhance the reliability of exercise tests. La at rest may not reflect CHF severity or exercise adaptation but correlates better with exercise capacity in CHF.

## 1 INTRODUCTION

Chronic heart failure is a complex syndrome characterized by progressive deterioration of ventricular function, resulting in decreased cardiac output and diminished functional capacity (Metra & Teerlink, 2017; Packer, 1992). Exercise intolerance is a major manifestation in patients with CHF (Okita et al., 2013). Several previous studies have focused on the importance of skeletal muscle anaerobic metabolism in limiting exercise capacity in this disorder (Poole et al., 2012; Wilson et al., 1984). For instance, Wever and Janicki have demonstrated that the early onset of La accumulation is an important predictor of submaximal exercise performance in CHF (Weber & Janicki, 1985).

It is well known that blood La concentration is increased at moderate and severe levels of muscular work, particularly in the venous blood (Davis, 1985; Margaria et al., 1933). This increase in venous La concentration, also called the La threshold, indicates the onset of blood La accumulation and presumably a shift to anaerobic metabolism in working skeletal muscle (Wasserman et al., 1973). At workloads above the La threshold, anaerobic metabolism predominates, and La production exceeds the rate of clearance and thus accumulates in the blood.

Exercise testing in animal models, particularly rodents with CHF, has been an important strategy for determining the presence and severity of cardiac dysfunction (Poole et al., 2020). Incremental treadmill running to exhaustion is commonly used to assess animals' aerobic exercise capacity (Billat et al., 2005; Kemi et al., 2002). Because maximal oxygen consumption linearly correlates with the maximal distance during an incremental exercise test, several studies utilize the maximal distance as an index of aerobic capacity (Ferreira et al., 2007; Poole et al., 2020). However, mice have different characteristics regarding energy metabolism during exercise. Thus, mice show variations in voluntary running distance and maximal running speed (Billat et al., 2005). The maximal distance achieved during an exercise test on a motorized treadmill is usually based on the maximal distance and speed that mice or rats can run before exhaustion. Exhaustion criteria, such as 10 consequent seconds on the electrical grid at the back of the treadmill and the inability to continue running despite manual prodding, have been used to gauge exhaustion during exercise tests (Erlich et al., 2018; Gibb et al., 2016). While these criteria may be useful in determining the maximal distance, they may be subjective. Measuring blood La when exhaustion criteria are met may provide an objective biochemical indicator of exhaustion at or near maximal oxygen consumption. Although the La threshold differs according to mice strain, the La threshold for C57BL/6J mice, a commonly used strain in experimental studies, ranges between 3.8 and 4.3 mM (Billat et al., 2005). Furthermore, it has been shown that on motorized treadmills, mice consistently struggle to run and become noncompliant when blood La is above 5 mmol/L, regardless of the absolute speed (Lønbro et al., 2019).

Exercise training induces several beneficial adaptations in CHF, including improved skeletal muscle oxidative capacity and peripheral oxygen extraction (Downing & Balady, 2011; Hirai et al., 2015). Thus, ExT delays a rise in blood La during exercise and lowers submaximal blood La levels (Sullivan, Higginbotham, & Cobb, 1989). In the rodent model of CHF focusing on exercise as a therapeutic tool, verifying exercise adaptations is extremely important for the potential efficacy of ExT. Ideally, direct measurements of oxygen consumption would provide a reliable measure of exercise adaptation (Poole et al., 2020); however, costs, personnel training, and operation are demanding. Although training adaptations can be assessed via ExT‐induced reduction of submaximal La, measuring blood La at submaximal intensities may be difficult and inconsistent due to different exercise test protocols used in rodents. Furthermore, measuring blood La at submaximal intensity may disrupt the rhythm of exercise test protocols. Therefore, although slightly invasive, resting blood La measurement is a simple and affordable method to assess training adaptations.

This study aimed to (1) evaluate the utility of blood La measurements at exhaustion as an objective biochemical indicator of fatigue during incremental exercise testing in CHF mice. We hypothesize that upon meeting exhaustion criteria, the blood La levels exceed the La threshold regardless of maximal speed; (2) assess whether resting blood La concentrations provide a reliable measure of CHF severity or exercise adaptation. We hypothesize that resting La levels are elevated in CHF‐Sed mice and reduced following ExT.

## 2 METHODS

### 2.1 Animal preparation

All animal procedures were approved by the Institutional Animal Care and Use Committee at the University of Nebraska Medical Center and adhered to the guidelines of the National Institutes of Health Guide for the Care and Use of Laboratory Animals.

**FIGURE 1:**
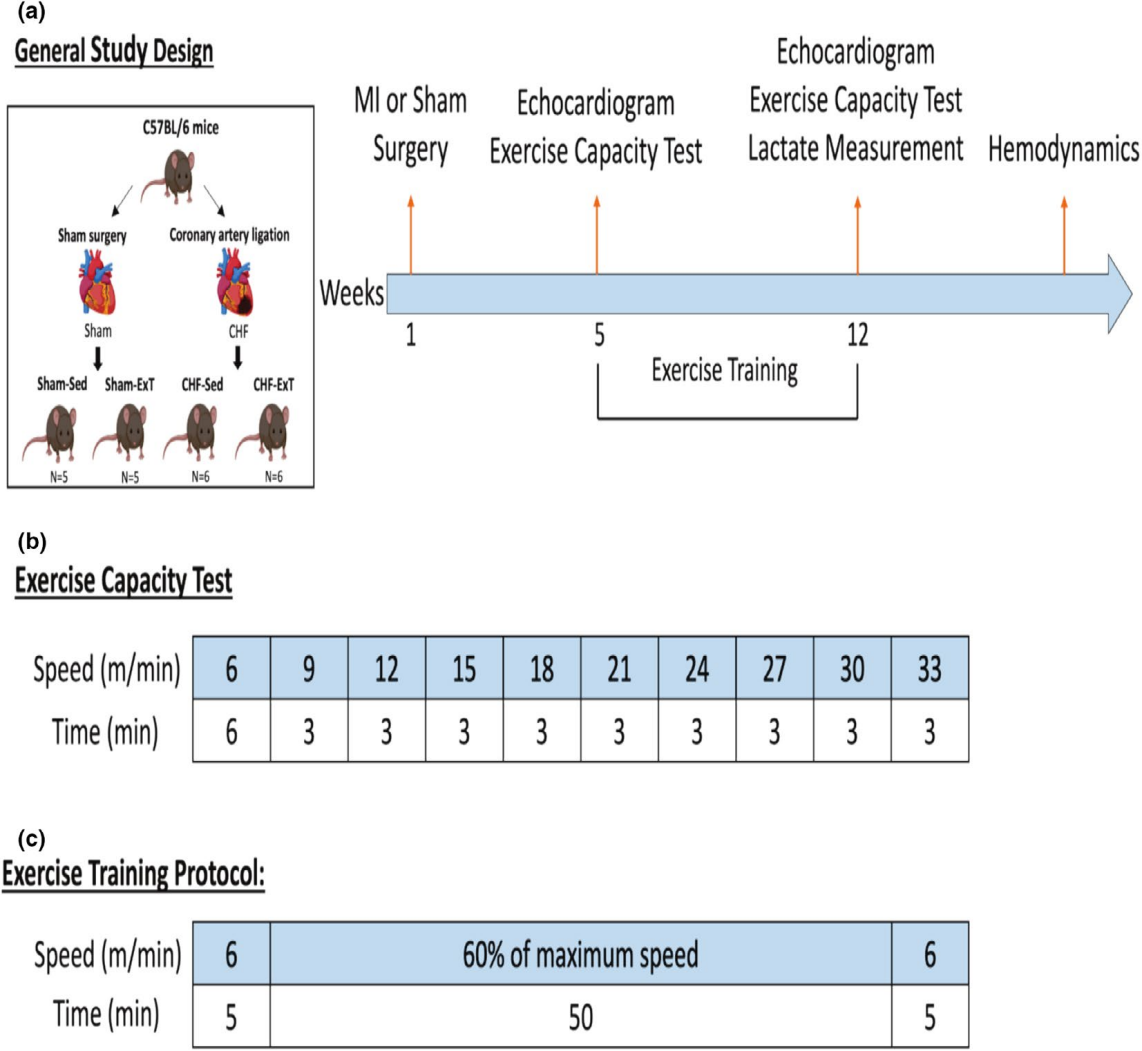
Schematics of the general study design (a), exercise capacity test (b), and exercise training protocol (c).

Figure 1a illustrates the general study design. Male C57BL/6J mice (8 weeks old) were purchased from Jackson Laboratory (Bar Harbor, ME) and allowed a 1‐week acclimation period under a controlled 12:12‐h light–dark cycle, with standard rodent chow (Envigo Teklad Global 18% Protein Rodent Diet, catalog #2018) and water provided ad libitum. Anesthesia was induced and maintained using inhalation of 2% isoflurane, and analgesia was provided by administration of buprenorphine (0.05 mg/kg) immediately following surgery and continued daily for three subsequent days. Heart failure was induced by ligation of the left anterior descending coronary artery, as previously described (Michael et al., 1995). Briefly, a left thoracotomy was performed at the fourth intercostal space, and the coronary artery was ligated permanently with a 7–0 suture in the CHF group. Sham‐operated mice underwent identical surgical procedures without ligation. After surgery, the chest and skin were closed using 6–0 and 4–0 sutures, respectively. Animals recovered on a heating pad maintained at 30–32°C and were provided supplemental oxygen (1 L/min) for 3 days postoperatively. Animals were then randomly assigned to one of four groups: Sham‐Sedentary (Sham‐Sed), Sham‐Exercise Trained (Sham‐ExT), CHF‐Sedentary (CHF‐Sed), and CHF‐Exercise Trained (CHF‐ExT). Infarct size was measured postmortem by tracing the scar area and the entire left ventricle (LV) with ImageJ software [National Institutes of Health (NIH), Bethesda, MD]. The percentage of the scar area relative to the total left ventricle was used to quantify the infarct size.

We selected male C57BL/6J mice because this strain is the most widely used background for chronic heart failure models induced by coronary ligation (Ahn et al., 2004; Gao et al., 2010; Ma et al., 2019). Although other strains, such as C57BL/6J, are known to exhibit better compliance with treadmill protocols compared to C57BL/6J, their metabolic phenotype lacks circadian variation, which may limit translational relevance (Gibb et al., 2016). To mitigate the impact of circadian rhythm disruption and to avoid interfering with exercise capacity, we conducted all exercise tests and exercise training sessions after 5 pm to minimize disruption of the nocturnal phenotype. Given that the C57BL/6J strain is commonly used in cardiovascular research, and treadmill running promotes physiological adaptations, such as cardiac hypertrophy and resting bradycardia in these mice after 4 weeks of exercise (Ferreira et al., 2007, 2010; Kemi et al., 2002), we believe that the characteristics of C57BL/6J mice are relevant and adequate for our study's aim of measuring blood La as a fatigue marker and assessing how exercise training affects resting La levels in CHF.

### 2.2 Echocardiography

Cardiac function was assessed by echocardiography 4 weeks following myocardial or sham surgery and following ExT using a VisualSonics Vevo 3100 system equipped with a 40 MHz transducer. The hair on the chests of the mice was removed using Nair (Church & Dwight Co., Inc., NJ, USA). Twenty‐four hours later, the mice were anesthetized with 1%–3% isoflurane (Cardinal Health, Dublin, OH, USA) and positioned supine on a heated pad set to 37°C. Anesthesia was maintained with 1%–2% isoflurane delivered through a nose cone. M‐mode images were acquired from parasternal long axis views to assess left ventricular end‐diastolic diameter (LVEDD), left ventricular end‐systolic diameter (LVESD), left ventricular anterior wall thickness‐diastole (LVAW;d), left ventricular anterior wall thickness‐systolic (LVAW;s), left ventricular posterior wall thickness‐diastolic (LVPW;d) and left ventricular posterior wall thickness‐systolic (LVPW;s), fractional shortening (FS), and percent ejection fraction (EF).

### 2.3 Hemodynamics measurement

Under 2% isoflurane anesthesia, the mice were placed in a supine position on a metal heating pad. The appropriate level of anesthesia was assessed by checking for the absence of reflex withdrawal in response to a painful stimulus, such as a foot pinch. The right common carotid artery was dissected, and a pressure transducer (SPR‐1000; Millar Instruments, Houston, TX) was advanced into the ascending aorta and left ventricle to measure blood pressure, left ventricular pressure, the first derivative of left ventricular pressure (dP/dt), and heart rate. The time constant of isovolumetric LV pressure fall (tau) was calculated using the method of Weiss et al. (1976). All signals were processed using a PowerLab data acquisition system (Model 8S) and analyzed with LabChart 7 software (ADInstruments, Colorado Springs, CO).

### 2.4 Exercise capacity test and exercise training

Maximal exercise capacity tests were performed on a motorized treadmill (Columbus Instruments, Columbus, OH) as previously described (Ferreira et al., 2007). The test was performed at two time points: 4 weeks post‐surgery and after the ExT regimen. Mice were familiarized with treadmill exercise over 3 days before the initial test, including a 10‐min session on a stationary treadmill with a mild shock (1.2 mA, 2 Hz) on day one and a 10‐min walking session at 6 m/min on day two and day three. As shown in Figure 1b, the incremental exercise test consisted of a 6‐min warm‐up at 6 m/min, followed by progressive increases in speed (3 m/min increments every 3 min) on a 5% incline until exhaustion. Exhaustion was defined as either spending five consecutive seconds on the shock grid without attempting to resume running or an inability to maintain treadmill speed consistently for 3 min (American Physiological Society, 2006). Distance run and peak speed achieved were recorded.

Mice assigned to the exercise‐trained groups underwent treadmill running 5 days per week for 8 weeks at approximately 60% of their individually determined maximal speed from the initial exercise capacity test. It has been previously shown that the La threshold occurred at 60% of the maximal speed achieved in the incremental exercise testing (Ferreira et al., 2007). Specifically, Sham‐ExT mice trained at approximately 14 m/min, while CHF‐ExT mice trained at around 11 m/min. Each training session began with a five‐minute warm‐up at 6 m/min, followed by running at the target training speed and a 5% incline. Sedentary mice were placed on a stationary treadmill two to three times weekly to control for handling and environmental exposure without undergoing actual exercise.

### 2.5 Blood lactate measurements

Blood La concentrations were measured using a La Plus analyzer (Nova Biomedical, Waltham, MA). Approximately 0.7 μL of blood was obtained from a tail vein prick immediately before exercise testing (baseline) and within 1 min after exhaustion. To minimize stress‐related variability in La levels, mice were familiarized with tail vein blood sampling prior to testing. In addition, before measuring La at rest, we physically restrained all mice in a plastic restrainer for 30 min to ensure they remained as inactive as possible.

Three days before blood sampling, all mice were restrained in a physical container for 30 min, followed by a tail punch to mimic the sampling procedure. Before measuring La at the baseline, mice were physically restrained to ensure all mice were physically inactive. Immediately after the exercise test, mice were restrained, and blood samples were drawn within 1 min.

### 2.6 Statistical analysis

Data are presented as mean ± SD, with statistical analyses conducted using GraphPad Prism (version 10.04). Differences between groups were analyzed by ordinary two‐way ANOVA. Paired *t*‐tests were used to compare blood La levels at baseline and exhaustion within each group. Spearman correlation analyses were used to explore relationships between baseline blood La levels, infarction size, and maximal exercise distance. Statistical significance was defined as *p* < 0.05.

## 3 RESULTS

### 3.1 Morphological and echocardiographic characteristics

**FIGURE 2:**
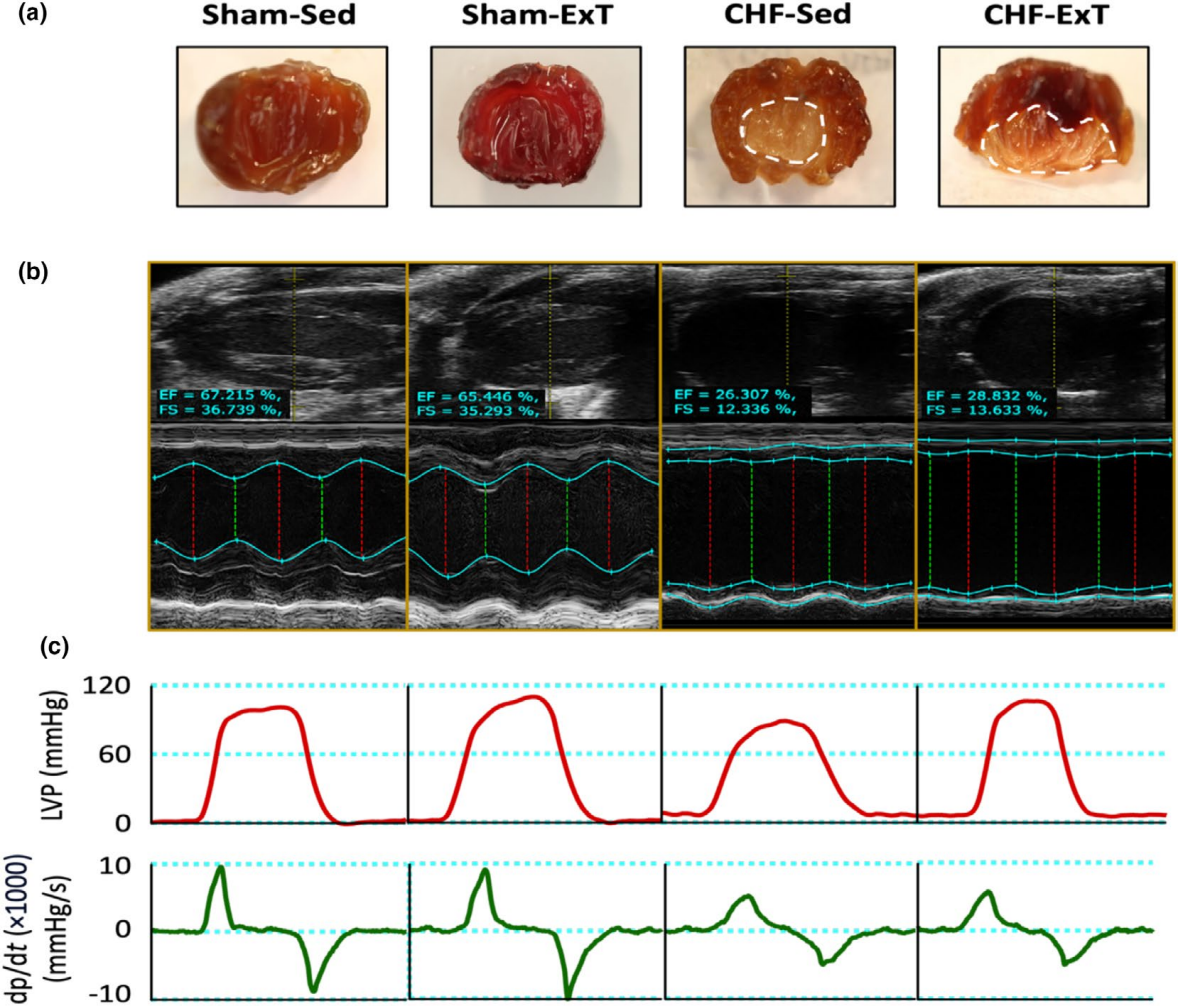
Representative infarct size, echocardiography images, and hemodynamics traces. (a) Example of a left ventricular infarct (dotted line) compared with a sham heart at 12 weeks after myocardial infarction. (b) Representative M‐mode and long‐axis echocardiography images of the left ventricle from each group. (c) Representative traces of left ventricular pressure and left ventricular dP/dt.

While there was no detectable damage to the sham mice hearts, CHF hearts showed marked infarcts (36.6 ± 5.9 in CHF‐Sed and 37.8 ± 4.1 in CHF‐ExT) (Figure 2a). Morphological and echocardiographic parameters of the experimental groups are presented in Table 1. CHF mice exhibited significantly increased heart weight, heart‐to‐body weight ratio, wet lung weight, left ventricular end‐systolic diameter (LVESD), left ventricular end‐diastolic diameter (LVEDD), left ventricular end‐systolic volume (LVESV), left ventricular end‐diastolic volume (LVEDV), left ventricular anterior wall diameter during diastole (LVAWd), left ventricular anterior wall diameter during systole (LVAWs), left ventricular posterior wall diameter during diastole (LVPWd), left ventricular posterior wall diameter during systole (LVPWs), and infarction size compared to Sham groups (*p* < 0.05). In addition, ejection fraction and fractional shortening were significantly reduced in CHF groups compared to sham groups (*p* < 0.001). Exercise training did not significantly alter these echocardiographic parameters in either Sham or CHF mice (Figure 2b).

**TABLE 1:** Morphological and echocardiography data.

	Sham‐sed (*n* = 5)	Sham‐Ext (*n* = 5)	CHF‐sed (*n* = 6)	CHF‐ExT (*n* = 6)
Body weight (g)	30.1 ± 0.8	30.1 ± 1.7	30.4 ± 2.2	30.9 ± 0.9
Heart weight (mg)	139.9 ± 4.5	155.5 ± 7.5	214.8 ± 46.2**	204.1 ± 28.6**
Heart weight/body weight (g/mg)	4.7 ± 0.2	5.1 ± 0.5	7.1 ± 1.3***	6.6 ± 1.0***
Wet lung weight (mg)	149.2 ± 9.8	152.1 ± 12.1	197.8 ± 43.9*	180.1 ± 19.0*
Wet lung weight/body weight (mg/g)	5 ± 0.3	4.9 ± 0.7	6.5 ± 2.6	5.7 ± 2.4
LVESD (mm)	2.3 ± 0.2	2.4 ± 0.4	5.3 ± 1.2***	4.4 ± 0.7**
LVEDD (mm)	3.6 ± 0.3	3.7 ± 0.4	6 ± 1.0***	5.1 ± 0.6**
LVESV (μL)	18.1 ± 4.7	20.6 ± 8.4	143.2 ± 78.9**	90.9 ± 35.0
LVEDV (μL)	55.2 ± 11.0	58.4 ± 15.4	183.4 ± 73.9***	127.7 ± 32.1
LVAW;d (mm)	1.1 ± 0.1	1.0 ± 0.2	0.6 ± 0.3*	0.7 ± 0.4
LVAW;s (mm)	1.4 ± 0.2	1.4 ± 0.2	0.8 ± 0.4	0.9 ± 0.8
LVPW;d (mm)	1.1 ± 0.1	1 ± 0.2	0.8 ± 0.3	1.0 ± 0.3
LVPW;s (mm)	1.3 ± 0.2	1.2 ± 0.2	0.9 ± 0.3	0.9 ± 0.4
HR (bpm)	443 ± 86.7	464 ± 59.1	403 ± 44.3	434 ± 48.6
EF (%)	67.6 ± 4.1	65.5 ± 4.8	25.5 ± 11.5***	28.3 ± 10.3***
FS (%)	36.4 ± 3.0	30 ± 3.1	15.8 ± 7.6***	16.7 ± 7.8***
IS (%)	0	0	36.6 ± 14.5***	37.8 ± 10.0***

*Note*: Data are presented as mean ± SD. Significance versus ShamSed is indicated by asterisks: *p < 0.05, **p < 0.01, ***p < 0.001.

Abbreviations: EF, ejection fraction; FS, fractional shortening; IS, infarction size; LVAW, left ventricular anteroir wall thickness; LVEDD, left ventricular end‐diastolic diameter; LVEDV, left ventricular end‐diastolic volume; LVESD, left ventricular end‐systolic diameter; LVESV, left ventricular end‐systolic volume; LVPW, left ventricular posterior wall thickness.

### 3.2 Hemodynamics

Hemodynamic data are summarized in Table 2. Left ventricular end‐diastolic pressure (LVEDP) was significantly higher in the CHF groups compared to the Sham groups (*p* < 0.01). CHF mice also exhibited significantly reduced maximal rate of pressure development (dP/dt max), minimal rate of pressure development (dP/dt min), and left ventricular systolic pressure (LVSP) compared to Sham controls (all *p* < 0.01). Exercise training did not significantly affect these hemodynamic parameters (Figure 2c).

**TABLE 2:** Hemodynamic data.

	Sham‐sed (*n* = 5)	Sham‐Ext (*n* = 5)	CHF‐sed (*n* = 6)	CHF‐ExT (*n* = 6)
LVEDP (mmHg)	1.9 ± 0.9	2.3 ± 0.6	6.9 ± 2.7***	6.1 ± 1.4**
dpdt max (mmHg/s)	9610.4 ± 615.1	10039.6 ± 801.2	4199 ± 1363.8***	5892.7 ± 1133.9***
dpdt min (mmHg/s)	−9742 ± 698.1	−9634 ± 947.5	−4023.5 ± 1222.1***	−5456.2 ± 1291.2***
LVSP (mmHg)	102.6 ± 1.8	99.3 ± 3.0	81 ± 13.2**	85.3 ± 10.5*
HR (bpm)	503 ± 22.4	470 ± 44.5	445 ± 53.6	510 ± 86.7
Tau (ms)	7.2 ± 0.5	7.7 ± 0.4	13.3 ± 5.4*	13.7 ± 2.5

*Note*: Data are presented as mean ± SD. Significance versus ShamSed is indicated by asterisks: *p < 0.05, **p < 0.01, ***p < 0.001.

Abbreviations: dP/dt max, peak positive first derivative of LV pressure; dP/dt min, peak negative first derivative of LV pressure; LVEDP, left ventricular end‐diastolic pressure; LVSP, left ventricular systolic pressure.

### 3.3 Exercise capacity test

**FIGURE 3:**
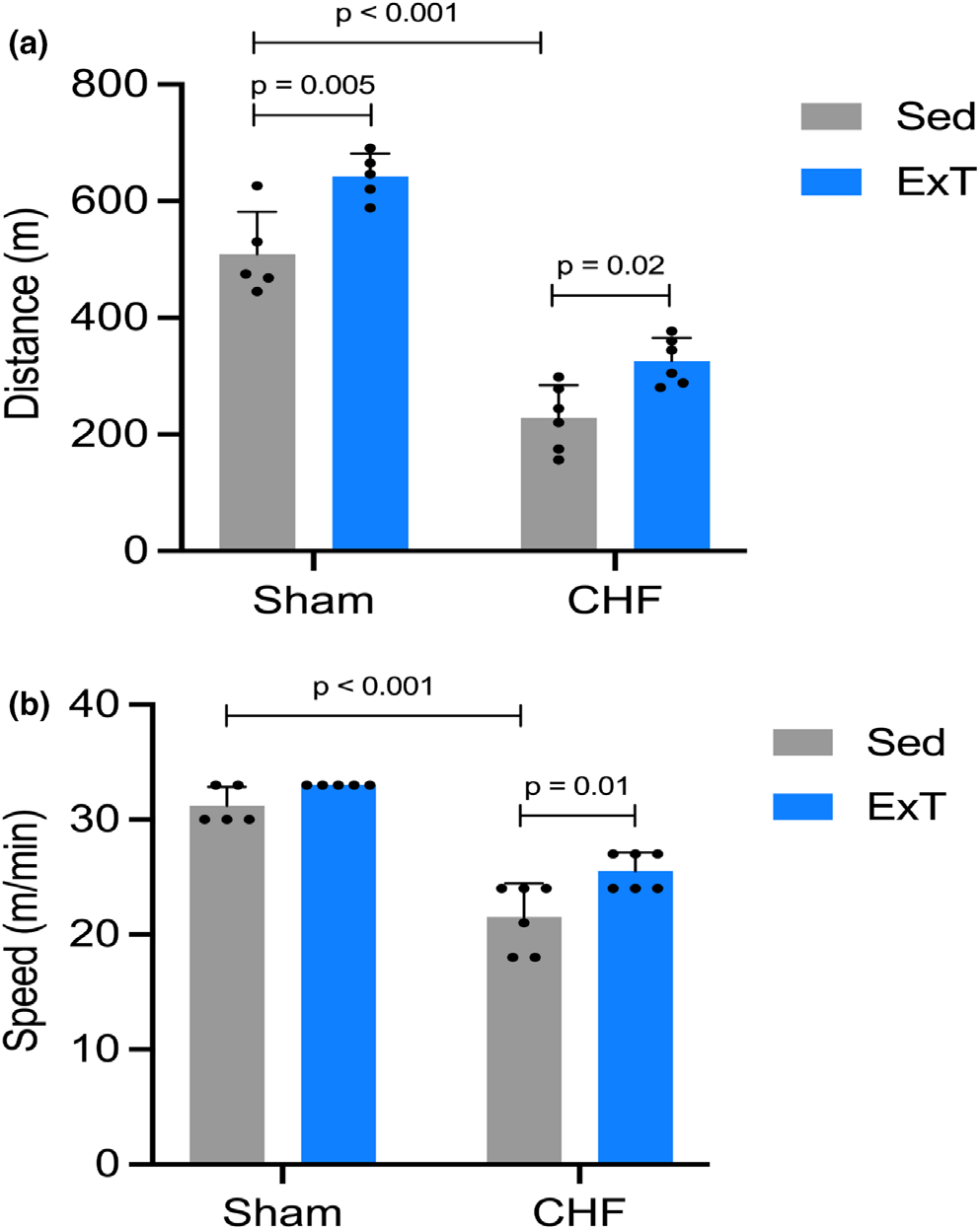
Maximal distance (a) and speed (b) achieved during the incremental exercise capacity test in Sham and CHF mice that were sedentary (SED) or exercise‐trained (ExT).

Maximal exercise capacity data are presented in Figure 3. CHF mice demonstrated significantly reduced maximal running distance and speed compared to Sham mice (*p* < 0.001). Exercise training significantly increased maximal running distance in both Sham (*p* = 0.005) and CHF mice (*p* = 0.02).

### 3.4 Blood lactate concentrations

**FIGURE 4:**
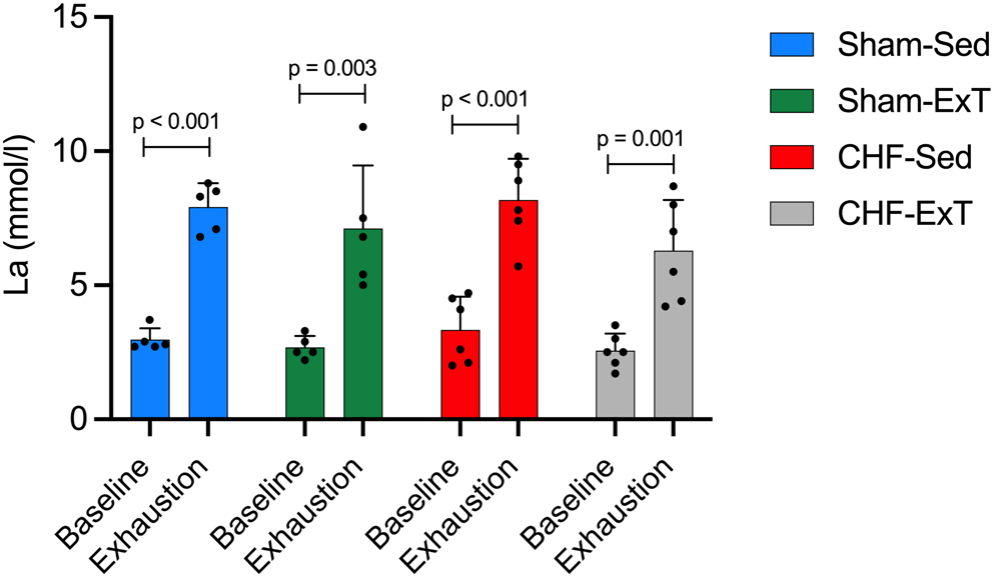
Blood La levels at rest and at exhaustion in sham and CHF mice that were sedentary (Sed) or exercise‐trained (ExT).

Blood La concentrations measured at baseline and exhaustion following the 8‐week ExT period are shown in Figure 4. Upon meeting the exhaustion criteria, blood La levels significantly increased in all groups compared to their respective baseline measurements (*p* < 0.01). Although baseline La was slightly elevated in CHF mice, there was no statistically significant difference in baseline La concentrations among the groups (Sham Sed: 2.96 ± 0.42 mmol/L, CHF‐Sed: 3.33 ± 1.23 mmol/L; *p* = 0.86).

**FIGURE 5:**
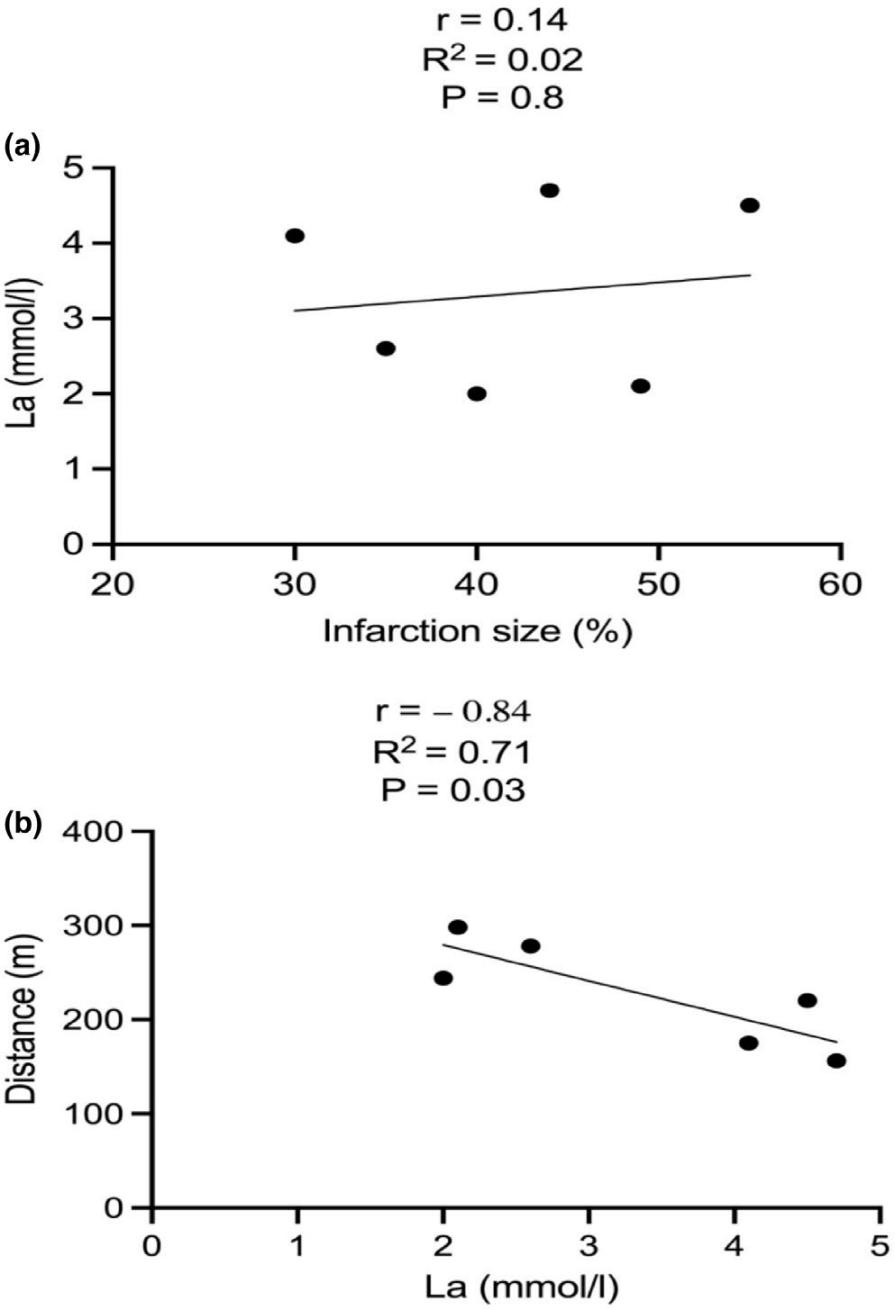
Correlation of (a) infarction size and baseline La in CHF‐Sed and (b) baseline La and maximal distance achieved during exercise capacity test in CHF‐Sed.

Spearman correlation analyses conducted in CHF sedentary mice revealed no significant correlation between baseline La concentrations and infarction size (Figure 5a) (*r* = 0.14, *R* ^2^ = 0.02, *p* = 0.8; Figure 5a). However, there was a significant negative correlation between baseline La concentrations and maximal running distance (Figure 5b) (*r* = −0.84, *R* ^2^ = 0.71, *p* = 0.03; Figure 4b).

## 4 DISCUSSION

This study evaluated the utility of baseline blood La as an indicator of heart failure severity and exercise adaptation in a CHF mouse model. In addition, this study examined whether blood La measured upon meeting exhaustion criteria during incremental exercise tests could serve as a biochemical indicator of maximal aerobic capacity by determining if blood La levels exceed the La threshold. Our results revealed impairments in echocardiographic and hemodynamic parameters and reduced exercise performance in CHF mice compared to Sham controls. Blood La levels increased significantly from baseline to exhaustion across all groups and were higher than the previously reported La threshold in C57BL/6J mice (Billat et al., 2005; Ferreira et al., 2007), suggesting that measuring La upon meeting exhaustion criteria during incremental exercise tests may provide an objective biochemical criterion for exercise‐induced fatigue.

Although baseline La was slightly elevated in CHF mice, no statistically significant differences were observed among groups. Nonetheless, our data revealed that in the CHF‐Sed mice, the baseline La negatively correlated with maximal running distance, suggesting that La at rest correlates more closely with exercise capacity in CHF mice than in Sham mice. Therefore, blood La at rest is a biomarker that may be used to predict exercise capacity in the CHF state. ExT improved running capacity in CHF mice despite the absence of improvements in cardiac function. In a previous study using an identical 8‐week ExT protocol, we observed a significant increase in citrate synthase activity in the soleus muscle of CHF mice (Wafi et al., 2019), suggesting that the improved exercise capacity may be partially attributed to peripheral adaptations. In addition, previous studies have suggested that improvements in skeletal muscle metabolism, endothelial function, and neurohormonal regulation are critical adaptations underlying enhanced aerobic capacity in response to ExT in the CHF state (Crimi et al., 2009; Pina et al., 2003; Wang et al., 2010).

An earlier study demonstrated that in healthy C57BL/6 mice, blood La levels were high upon meeting the exhaustion criteria during the maximal exercise test (Gibb et al., 2016). Our current findings extend this observation to mice with CHF, showing similarly elevated La levels at exhaustion, although at reduced workloads. While the treadmill is reliable and may be a superior tool for evaluating exercise capacity in research studies in rodent models (American Physiological Society, 2006), there are varieties of factors that contribute to early cessation of running before maximal exercise capacity is attained; these factors include mouse strain differences, testing environment, acclimatization procedures, motivation, and subjective assessment of exhaustion criteria (Lønbro et al., 2019; Poole et al., 2020). Thus, measuring La levels at the point of exhaustion during incremental exercise tests offers a minimally invasive approach that can enhance the reliability of exercise capacity assessments by ensuring that the inability of mice to continue running is more likely due to exhaustion. Therefore, measuring blood La at exhaustion can improve the reproducibility and validity of incremental exercise tests, regardless of the exercise test protocols and mouse strain employed.

The finding that blood La concentrations at exhaustion are similar between CHF mice and sham mice contrasts with previous human studies, which reported lower peak La levels in CHF patients due to lower achieved workloads (Sullivan, Knight, et al., 1989). While La kinetics differ between humans and mice (Fuller & Thyfault, 2021), this discrepancy may be due to exhaustion criteria employed in our study. Although CHF mice reach exhaustion at lower speeds and shorter distances, they likely reach their maximal exercise capacity limits at lower workloads due to reduced cardiorespiratory fitness and impaired muscle metabolism. Consequently, the La levels at exhaustion reflect the maximal effort relative to each mouse's individual capacity rather than the absolute workload achieved during the test.

There is conflicting evidence regarding resting La levels in heart failure patients compared to healthy individuals, with some studies showing no significant differences (Sullivan et al., 1991; Sullivan, Knight, et al., 1989; Yamabe et al., 1994), while other studies report higher resting La levels in CHF patients, particularly in those with the lowest functional capacity (Andrews et al., 1997; Schaufelberger et al., 1996; Wilson et al., 1984). High blood La level is a marker of poor prognosis in patients with heart failure (Zymliński et al., 2018). While we observed a trend toward a higher resting La in CHF‐Sed mice, we found no significant differences in the resting La levels between the CHF‐Sed and Sham‐Sed mice. Nonetheless, our finding of a negative correlation between the baseline La level and running distance aligns with human findings of an early switch to anaerobic metabolism, which limits exercise capacity (Sullivan & Cobb, 1990).

Contrary to our hypothesis, the baseline blood La levels were not elevated in CHF‐Sed mice compared with sham‐controlled. This finding may be attributed to the inherently high vascular sympathetic tone in mice, which could limit the sympathetic tone reserve (Janssen & Smits, 2002). This limited reserve might not substantially impair oxygen delivery following myocardial infarction, potentially preventing a significant rise in resting blood La levels. Alternatively, the infarction size in our study might not have been large enough to sufficiently alter metabolism and La accumulation in the blood. Smaller or moderate‐sized infarctions may allow mice to maintain adequate cardiac output and tissue perfusion at rest, thereby preventing substantial increases in La production at baseline. Consistent with this, a previous study by Sullivan, Knight, et al. (1989) found that while heart failure patients exhibit higher La levels during submaximal exercise, resting La levels are generally similar to those in healthy individuals. However, patients with lower functional capacity due to more advanced heart failure display elevated La levels at rest. Another possibility for the lack of elevated blood La levels in CHF‐Sed mice is that the timing of La measurement (12 weeks post‐MI) may have allowed several metabolic adaptations to occur. Early after myocardial infarction, La concentrations might be substantially higher due to acute hypoxia and increased anaerobic metabolism (Uyar et al., 2020; Zymliński et al., 2018). However, over time, compromised coronary arteries are partially compensated by collateral blood flow, resulting in normal myocardial blood flow and contractile function at rest (Jamaiyar et al., 2019; Lindsey et al., 2018).

While the blood La level at rest was not different among the groups, our findings revealed a significant negative correlation between the blood La at rest and maximal distance achieved during incremental exercise tests in CHF‐Sed mice. In CHF, there is an early onset of skeletal muscle acidosis and La production, often measured as an increase in venous La at a given workload, resulting in early anaerobic metabolism and reduced exercise capacity (Sullivan & Cobb, 1990; Weber & Janicki, 1985). Although we did not evaluate the blood La kinetics during the exercise capacity test, the observed negative correlation between resting blood La and maximal distance in CHF‐Sed mice suggests that even slight elevation in resting La not only predicts exercise capacity but also may be a sensitive marker of the peripheral metabolic alterations such as impaired oxidative metabolism, reduced mitochondrial function, and peripheral vasoconstriction that limit exercise capacity in CHF.

The modest trend toward lower resting La in the CHF‐ExT group may reflect metabolic adaptations, including mitochondrial oxidative capacity, consistent with training‐induced improvements in skeletal muscle metabolism (Holloszy & Booth, 1976). However, the complex metabolic milieu of heart failure, characterized by impaired cardiac output and peripheral perfusion, may attenuate the magnitude of these adaptive responses (Holloszy & Booth, 1976; Wafi et al., 2019). In addition, the relatively small number of mice in our study may have diluted the effect of exercise training on resting blood La, and it is possible that with a larger sample size, a statistically significant reduction might be observed. While our findings suggest that resting La may not serve as a highly sensitive marker of training adaptation in CHF with the current sample size, the negative correlation between baseline La and exercise capacity indicates that this simple, cost‐effective measurement retains prognostic value for assessing functional capacity in heart failure models.

Our study has several limitations; the small number of animals, in part due to high mortality rates during and after myocardial infarction surgery, may have reduced our ability to detect a meaningful difference in La measurements between the sham and the CHF groups. Although the mice were acclimated to treadmill electrical stimulation, forced treadmill running via electrical shock grids may still induce stress, potentially altering metabolic parameters, including blood La levels, and impacting exercise performance (Svensson et al., 2016). In addition, we did not conduct a test–retest measurement to enhance the validity of La measurement. Furthermore, we did not measure maximal oxygen uptake, which would have provided a more accurate assessment of exercise‐induced fatigue, although strict exhaustion criteria were employed during exercise testing. The use of the C57BL/6J mouse strain may not be optimal for assessing exercise capacity or exercise adaptations. Previous research indicates that, unlike other strains, such as FVB/NJ, C57BL/6J mice exhibit poorer compliance with treadmill ExT protocols (Erlich et al., 2018), potentially influencing our results related to exercise performance and adaptation. Furthermore, we did not measure the La threshold, nor did we measure the maximal La steady state. We also did not assess mitochondrial function, fiber type shifts, or enzymatic changes such as citrate synthase activity.

Future studies should reduce stress when measuring blood La levels. For example, measuring blood La under anesthesia could minimize stress effects and improve measurement accuracy. In addition, future studies should examine the changes in blood La levels before and after an ExT period to better understand changes in blood La in response to ExT. Measuring blood La at different time points following myocardial infarction along with aerobic exercise capacity could also provide insights into the relationship between resting La metabolism and exercise performance. Furthermore, examining the relationship between infarct size (small, medium, and large) and basal blood La levels would further clarify how myocardial injury severity influences basal blood La levels.

## AUTHOR CONTRIBUTIONS

A.W., I.Z., and L.G. conceived and designed research, interpreted results of experiments, edited and revised manuscript, and approved final version of the manuscript. A.W. and L.G. performed experiments. A.W. analyzed data, prepared figures, and drafted manuscript.

## FUNDING INFORMATION

This project was supported by a grant from the National Heart, Lung, and Blood Institute, P01 HL‐62222. This work was conducted as part of Ahmed Wafi's doctoral dissertation at the University of Nebraska Medical Center.

## CONFLICT OF INTEREST STATEMENT

No conflicts of interest, financial or otherwise, are declared by the author(s).

## ETHICS STATEMENT

All animal experiments were approved by the Institutional Animal Care and Use Committee of the University of Nebraska Medical Center and were carried out under the guidelines of the National Institutes of Health Guide for the Care and Use of Laboratory Animals.

## Data Availability

The data for this study are available from the corresponding author upon reasonable request.
